# Exploring the best treatment options for BRAF-mutant metastatic colon cancer

**DOI:** 10.1038/s41416-019-0526-2

**Published:** 2019-07-29

**Authors:** Julien Taieb, Alexandra Lapeyre-Prost, Pierre Laurent Puig, Aziz Zaanan

**Affiliations:** 1 Sorbonne Paris-Cité, Paris Descartes University, Assistance Publique Hôpitaux de Paris (APHP), Gastro-enterology and GI Oncology Department, Georges Pompidou European Hospital, Paris, France; 2 INSERM UMR-S1138, CNRS SNC5014, Paris Descartes University, Equipe labellisée Ligue Nationale contre le Cancer, Paris, France; 3 Sorbonne Paris Cité, Paris Descartes University, Assistance Publique Hôpitaux de Paris, Department of Biology, Georges Pompidou European Hospital, Paris, France

**Keywords:** Colon cancer, Colon cancer

## Abstract

The BRAF^V600E^ mutation is a well-accepted poor prognostic factor in patients with metastatic colorectal cancer (mCRC), as it confers Ras-independent stimulation of the extracellular signal-regulated kinase/mitogen-activated protein kinase pathway involved in proliferation, migration, angiogenesis and the suppression of apoptosis. Analysis of the potential predictive value of *BRAF* for treatment efficacy is inherently confounded by this known prognostic impact. Currently, approved therapeutic strategies for patients with *BRAF*-mutant (*BRAF*-mt) mCRC are suboptimal, and uncertainty exists regarding how to best treat these patients. Based on the available evidence, it is currently not possible to confirm the superiority of any available treatment options cited in European Society for Medical Oncology and National Comprehensive Cancer Network guidelines (that is, doublet or triplet chemotherapy regimens plus anti-vascular endothelial growth factor or anti-epidermal growth factor receptors), even if triplet chemotherapy plus bevacizumab is the most accepted standard regimen. In this review, we highlight still-emerging strategies that could be deployed to combat *BRAF*-mt mCRC, including triplet chemotherapy plus available biologic agents, rationally derived combinations of targeted agents and immunotherapy. While it is clear that the needs of patients with *BRAF*-mt mCRC are currently unmet, we are cautiously optimistic that the recently renewed research interest in these patients will yield clinically relevant insights and therapeutic strategies.

## Background

Despite significant progress in the treatment of colorectal cancer (CRC) over the past 15 years, the disease remains a leading cause of cancer-associated mortality worldwide.^1^ During the past decade, molecular testing in patients with metastatic CRC (mCRC) has become standard practice, and knowledge of *RAS*, *BRAF* and microsatellite instability (MSI) status is nowadays mandatory if we are to offer patients the best treatment and has contributed to the improved clinical outcome for patients with mCRC.^2^ Although it has been known since 2014 that mCRC caused by mutated *RAS* is resistant to anti-epidermal growth factor receptor (EGFR) therapy^3,4^ and since 2015 that the MSI phenotype is sensitive to immunotherapeutic agents,^5,6^ CRC patients with a mutation in *BRAF* are still awaiting a specific and tailored therapeutic approach. BRAF is a key downstream effector of RAS in the mitogen-activated protein kinase (MAPK)/extracellular signal-regulated kinase (ERK) signal transduction pathway, which mainly influences cell proliferation, differentiation and apoptosis. *BRAF* is therefore considered to be an oncogenic driver in colorectal tumours,^7^ although the molecular, morphological, epidemiological and clinical characteristics of the serrated polyps initiated by *BRAF* differ from the polyps of the ‘classic’ adenoma–carcinoma sequence driven by mutations in the adenomatous polyposis coli (*APC*) gene.^8^

*BRAF* mutations, which are thought to be mutually exclusive of *RAS* mutations, arise in 5–10% of patients with mCRC.^9^ However, the prevalence of *BRAF* mutations might be underestimated because patients with these mutations are often ineligible for enrolment in clinical trials owing to their poor performance status and age. Indeed, the prevalence of *BRAF* mutations was recently reported to be as high as 21% in CRC patients in a Norwegian registry.^10^ The overwhelming majority (> 95%) of *BRAF* mutations in mCRC occur in codon 600, involving a T1799A transversion in exon 15, which results in the substitution of a valine amino acid for a glutamic acid (V600E mutation). Non-V600E *BRAF* mutations occur in ~2% of patients with mCRC and define a clinically distinct subtype with a better prognosis.^11,12^ Indeed, in a recent retrospective analysis of 2084 mCRC patients, overall survival (OS) was 39.4 months in patients with non-V600E BRAF mutations, whereas it was only 21 months in ^V600E^BRAF-mutant patients. However, efficacy of anti-EGFR seemed limited in this cohort for non-V600E BRAF-mutant and RAS wild-type patients, and the predictive impact of these rare mutations remains unknown so far.^13^ *BRAF*-mt CRC used throughout this article will thus refer exclusively to the V600E mutation.

Gene-expression profiling studies have established that BRAF^V600E^-mutant *BRAF*-mt CRC is enriched in a molecularly and clinically distinct disease subtype, which is frequently associated with hypermethylation, MSI, limited chromosomal instability, consensus molecular subtype 1, a higher rate of recurrence in an adjuvant setting and poor survival outcomes in the metastatic setting.^14,15^

Numerous studies have confirmed the prognostic relevance of *BRAF* mutational status for both localised and metastatic colon cancers: patients with *BRAF*-mt CRC have impaired survival (Table 1) not only in the metastatic setting but also in non-metastatic disease as compared with patients with *BRAF* wild-type (*BRAF*-wt) CRC.^11,16–18^ Indeed, according to a meta-analysis of 11,321 patients, the risk of death was more than doubled in patients with *BRAF*-mt compared with those with *BRAF*-wt disease.^19^ Current therapeutic strategies, with doublet or triplet chemotherapies plus a targeted agent, for mCRC have achieved median OS exceeding 30 months in randomised phase 3 clinical trials involving patients with *RAS* wild-type mCRC,^20,21^ and 25 months in mCRC patients not selected for their *RAS* status.^22^ A recent meta-analysis restricted to patients with *KRAS*-wt mCRC reported significantly impaired survival in patients with *BRAF*-mt/KRAS wt disease, with a median OS of 10.8 months.^23^

**Table 1:** Prognostic impact of *BRAF*-mutation in randomised clinical trials and retrospective studies

CRC stage	Reference	*BRAF*-mt/total (%)	*BRAF*-mt/MSI (%)	*BRAF*-mt/MSS (%)	Impact on survival	
I–IV (retrospective cohorts)	72	87/911 (10)	43/83 (52)	40/803 (5)	OS	Negative, except MSI (no impact)
	66	182/1253 (15)	101/193 (52)	81/1060 (8)	CRC-specific mortality	Negative
II–III	73	103/1307 (8)	45/188 (24)	53/1055 (5)	RFS	No impact
					OS	Negative, except MSI (no impact)
	74	316/2299 (14)	71/207 (34)	176/1589 (11)	SAR	Negative
					OS	
	75	94/902 (10)	20/85 (24)	74/817 (9)	OS	No impact
III	76	346/2515 (14)	150/314 (49)	190/2266 (9)	DFS	Negative
	11	148/1643 (9)	54/177 (32)	94/1614 (6)	DFS	Negative, except MSI
					OS	
Metastatic CRC	65	250/3063 (8)	53/153 (35)	197/ (7)	PFS	Negative
					OS	
	28	127/1567 (8)	–	–	PFS	Negative
	77	74/664 (11)	–	–	PFS	Negative
					OS	
	15	480/4411 (11)	201/477 (42)	279/3934 (7)	OS	Negative
					TTR	
	78	100/843 (12)	–	–	OS	Negative

*CRC* colorectal cancer, *OS* overall survival, *RFS* relapse-free survival, *SAR* survival after resection, *DFS* disease-free survival, *PFS* progression-free survival, *MSI* microsatellite instability, *TTR* time to progression

Given this poor outcome in patients with *BRAF*-mt mCRC, the optimisation of therapy is an important goal. In this review article, we summarise current treatment options for patients with *BRAF*-mt mCRC, as well as emerging strategies that, taken together, show the continued need for additional dedicated studies in these patients.

## The BRAF pathway

**Fig. 1:**
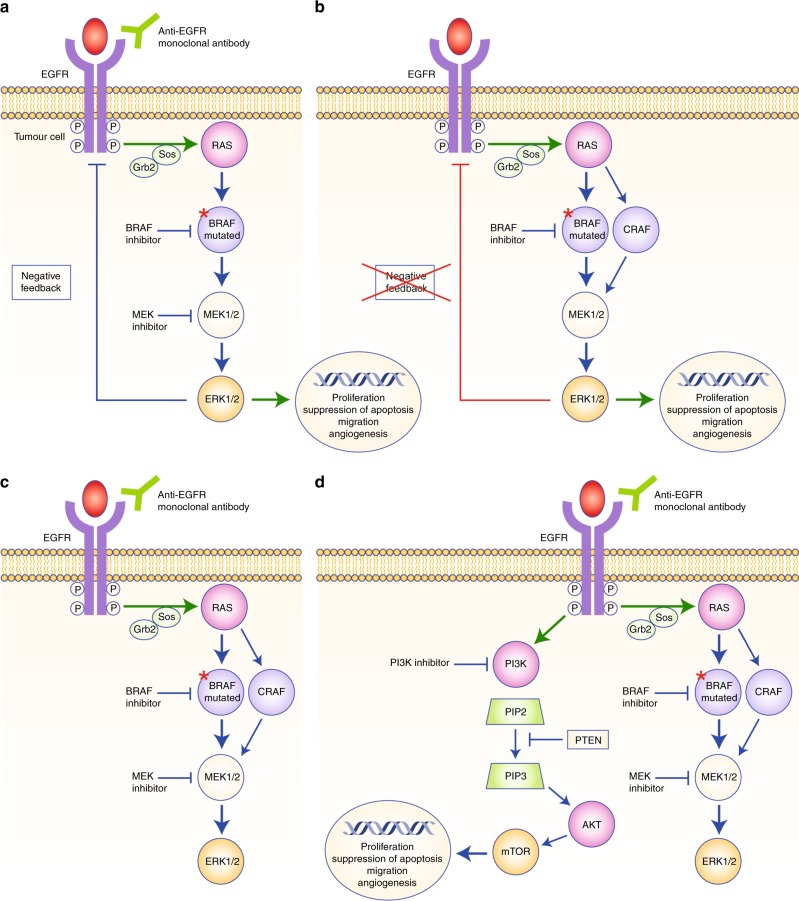
The BRAF pathway. **a** Activated BRAF-mutated protein leads to phosphorylation and activation of mitogen-activated protein kinase (MAPK)/extracellular signal-regulated kinase (ERK) kinase (MEK)1/2 proteins, which subsequently phosphorylate and activate ERK1/2 proteins. After activation, ERK proteins phosphorylate a variety of substrates, including multiple transcription factors and regulate several key cellular activities, such as proliferation, differentiation and angiogenesis, to promote tumour growth. **b** Inhibition of BRAF suppresses the ERK-mediated negative feedback of the epidermal growth factor receptor (EGFR), resulting in EGFR activation, formation of RAF protein dimers and CRAF-mediated reactivation of the MAPK signalling pathway. **c** Preclinical studies have shown efficacy with combination drugs targeting BRAF (BRAF inhibitor), MEK (MEK inhibitor) and EGFR (anti-EGFR monoclonal antibody); this triplet combination might be an interesting therapeutic approach in patients with BRAF-mutated mCRC. **d** Crosstalk between the RAS/BRAF/MEK/ERK and the PI3K/AKT/mammalian target of rapamycin (mTor) signalling pathways after BRAF inhibition could play a determinant role in cell survival. Combining BRAF, EGFR and PI3K inhibitors could constitute another interesting therapeutic approach in patients with BRAF-mutated mCRC

The RAS/MAPK pathway, together with the PI3K (phosphatidylinositol 3-kinase)/AKT pathway, constitutes one of the best-known signal transmission pathways resulting, after a cascade of successive phosphorylations, in the transcription of genes involved in cancer development. The MAPK/ERK signalling cascade conveys mitogenic and other stimulatory signals from receptors, such as EGFR, on the cell membrane to the nucleus. Activation of the RAF family of serine/threonine kinases proteins by a Ras small guanidine triphosphatase (GTPase) downstream of cell–surface receptors leads to the phosphorylation and activation of MAPK and ERK kinase (MEK)1/2 proteins, which subsequently phosphorylate and activate ERK1/2 proteins. Upon activation, ERK proteins phosphorylate a variety of substrates, including multiple transcription factors, and regulate several key cellular activities, such as proliferation, migration, angiogenesis and the suppression of apoptosis (Fig. 1a). The RAF family also includes ARAF (also known as ARAF1) and CRAF (also known as RAF1), but BRAF has the strongest basal kinase activity and is the most potent activator of MEK/ERK proteins.

Aberrant signalling or inappropriate activation of the MAPK/ERK signalling pathway is involved in many human malignancies.^24^ Among solid tumours, the highest prevalence of activating somatic missense *BRAF* mutations, with the V600E substitution accounting for ~80% of mutations, occurs in malignant melanomas (60–70%); mutations occur at a lower frequency in other human cancers, such as papillary and anaplastic thyroid carcinomas (40–50%), ovarian (30%) and CRCs (10–20%). All mutations in BRAF confer increased kinase activity compared with the wild-type protein, and thereby stimulate MAPK/ERK activity in a Ras-independent manner.^7^

Finally, *BRAF*-mt patients should not be considered as having a unique biology. In fact, Barras et al.^25^ have even recently described, from a series of 218 *BRAF*-mt patients with colon cancer, two distinct subtypes of patient, independent of their gender, primary tumour location, mismatch repair (MMR) status and PI3K status. The BM1 subtype, representing one-third of patients, is associated with the strong activation of AKT/mammalian target of rapamycin (mTOR), KRAS, 4EBP1 and epithelial–mesenchymal transition features, whereas BM2, representing the remaining two-thirds of *BRAF*-mt patients, displays deregulation of the cell cycle, with high levels of cyclin-dependent kinase (CDK)1 and low cyclin D1.

BRAF inhibitors have proven clinical activity in *BRAF*-mutant patients in other tumour locations, such as melanoma, but resistance emerges frequently due to a multitude of escape mechanisms, thereby necessitating combination treatment (Fig. 1b). For instance, in vitro studies have suggested that BRAF inhibition suppresses ERK-mediated negative feedback on EGFR activity, resulting in EGFR activation, the formation of RAF protein dimers and CRAF-mediated reactivation of the ERK/MAPK signalling pathway. Thus, EGFR signalling seems to play a critical role in bypassing BRAF inhibition and mediating therapeutic resistance.^26,27^ This may explain partly the insufficient efficacy of anti-BRAF monotherapy since BRAF inhibition induces a simultaneous overexpression of EGFR receptor leading on the one hand to the adaptive feedback reactivation of MAPK signalling via CRAF and on the other hand to the activation of the PI3K/AKT pathway, which also depends on the EGFR signal. To overcome these resistance mechanisms, blocking both BRAF, EGFR and MEK (Fig. 1c) and combining BRAF and PI3K inhibitors make sense (Fig. 1d).

## Current systemic treatments for *BRAF*-mt mCRC

Current standard first-line chemotherapy for mCRC patients involves the combination of a fluoropyrimidine and either irinotecan or oxaliplatin. Standard chemotherapy has been evaluated in a retrospective cohort of 127 *BRAF*-mt mCRC patients, and has shown very poor outcomes in terms of progression-free survival (PFS) for the first three lines of chemotherapy (median PFS of 6.3, 2.5 and 2.6 months, respectively). The choice of systemic therapy used (oxaliplatin-based or irinotecan-based regimen) did not significantly affect PFS in first-line treatment (6.4 versus 5.4 months, *P* = 0.99).^28^

A more aggressive strategy, involving combination of doublet with the EGFR inhibitor, cetuximab, or triplet with bevacizumab, which inhibits vascular endothelial growth factor (VEGF), might be of interest in mCRC patients with *BRAF*-mt tumours, as suggested by some clinical data, although these data are based on small subgroups.^17,29–32^ Indeed, in patients with *KRAS*-wt/*BRAF*-mt mCRC included in the CRYSTAL randomised trial comparing FOLFIRI (folinic acid, fluorouracil and irinotecan) alone or combined with cetuximab, a trend to improvement in both PFS (median, 8.0 versus 5.6 months; HR, 0.93; *P* = 0.87) and OS (median, 14.1 versus 10.3 months; HR, 0.91; *P* = 0.74) was observed in favour of the anti-EGFR monoclonal antibody-based treatment.^17^ In a 2010 study by Masi et al.,^29^ patients with wild-type and mutated *BRAF* CRC tumours had similar median PFS and OS when the treatment was based on the triplet FOLFOXIRI (folinic acid, fluorouracil, oxaliplatin and irinotecan) with bevacizumab, suggesting that this aggressive therapeutic strategy could also lead to the loss of the negative prognostic impact of *BRAF* mutation. Loupakis et al.^30^ conducted one of the first phase 2 studies conceived to explore an intensified regimen with FOLFOXIRI plus bevacizumab specifically in *BRAF*-mt patients (15 patients) and showed interesting results (median OS, 19 months; median PFS, 7.5 months). Finally, in a randomised phase 3 trial comparing bevacizumab plus FOLFIRI to bevacizumab plus FOLFOXIRI in mCRC patients, the subgroup of *BRAF*-mt patients appeared to benefit from the addition of oxaliplatin in terms of OS [19 versus 10.7 months; HR, 0.54 (95% CI, 0.24–1.20)], and PFS [7.5 versus 5.5 months; HR, 0.57 (95% CI, 0.27–1.23)], although this survival benefit did not reach statistical significance.^22,31^

These results led to the recommendation of this upfront, aggressive schedule in patients with *BRAF*-mt mCRC in most recent guidelines.^2,33,34^ However, it is important to emphasise that this ‘standard treatment’ is based on the observation of fewer than 100 patients in three studies. However, even if the level of evidence remains weak, this strategy is well accepted because it offers an aggressive upfront treatment, including all major chemotherapeutic agents for mCRC and a targeted therapy, with a manageable toxicity profile, to treat patients with a particularly aggressive disease who are rarely able to receive a second-line treatment.

## Efficacy of registered targeted agents

### Anti-angiogenic agents

Although it has been shown that the MAPK signalling cascade can increase VEGF expression and that *BRAF* mutation might also modulate tumour response to anti-angiogenic treatments,^35^ the value of bevacizumab in *BRAF*-mt patients has not yet been clinically demonstrated. In fact, in the previously reported results, although the addition of oxaliplatin to FOLFIRI plus bevacizumab treatment seemed beneficial over FOLFIRI plus bevacizumab treatment, the added value of the anti-angiogenic agent has not been shown.^31^ However, even if no randomised data evaluating the influence of adding bevacizumab to standard chemotherapy (i.e., FOLFIRI or FOLFOX) are available from patients with *BRAF*-mt mCRC, the addition of bevacizumab to first-line IFL [bolus irinotecan, fluorouracil and leucovorin (folinic acid)] or capecitabine has shown a numerical improvement in survival outcomes in patients with *BRAF*-mt mCRC in post-hoc analyses of the AVF2107g^35^ and AGITG MAX^36^ trials. In addition, the results of the VELOUR trial biomarker analysis^37^ have recently been reported. The corresponding clinical trial randomised aflibercept [a fusion protein that binds circulating VEGF-A, VEGF-B and placental growth factor (PlGF)] versus placebo, in combination with FOLFIRI chemotherapy, in second-line treatment. For the biomarker analysis, 482 samples were collected from 1226 randomised patients (39% of the patients) with mCRC who progressed after oxaliplatin-based first-line chemotherapy. The results showed that the *BRAF*-mutated population (*n* = 36, 7.5%) benefitted more from addition of aflibercept [OS HR, 0.42, (95% CI, 0.16–1.09)] than did the *BRAF*-wt population, but the difference was not significant [HR, 0.49 (95% CI, 0.22–1.09), *P* = 0.08], probably due to the small series of patients. Similar results were reported with the RAISE trial biomarker analysis^38^ using FOLFIRI in second-line treatment with another anti-angiogenic agent, ramucirumab, that targets VEGFR2. Although these post-hoc analyses of randomised trials suggest that anti-angiogenic agents might be of interest in *BRAF*-mt mCRC patients, prospective trials comparing an aggressive chemotherapy alone or in combination with an anti-angiogenic therapy are still awaited.

### Anti-EGFR agents

Concerning anti-EGFR agents, current data and publications are confusing. Nevertheless, it seems quite obvious that anti-EGFR monoclonal antibodies (panitumumab and cetuximab) provide no benefit for *BRAF*-mt mCRC patients when these therapies are used as single agents in patients heavily pre-treated with chemotherapy.^39^ Similarly, in second-line treatment, two studies evaluating the addition of anti-EGFR to FOLFIRI have reported the same results, with no clinical benefit to *BRAF*-mt mCRC patients.^40,41^ The PICCOLO trial even reported a deleterious effect, in terms of OS [HR, 1.84 (95% CI, 1.10–3.08), *P* = 0.029], of adding panitumumab to irinotecan treatment in patients with *BRAF*-mt tumours.^41^

The results of first-line treatment using the combination of chemotherapy plus anti-EGFR agents are less clear. The pooled analysis data of CRYSTAL and OPUS randomised studies evaluating the addition of cetuximab to first-line FOLFIRI or FOLFOX chemotherapy in *KRAS*-wt mCRC patients have shown an improvement of objective response rate (ORR), PFS and OS in the subgroup of *BRAF*-mt mCRC patients.^32^ The authors concluded that the *BRAF* mutation does not appear to be a predictive biomarker of resistance to anti-EGFR therapy in this setting, only a marker of poor prognosis. Similarly, the addition of panitumumab to FOLFOX first-line chemotherapy was associated with a numerical improvement of efficacy outcomes in the *KRAS*-wt/*BRAF*-mt subgroup.^4^

Two meta-analyses have been performed on the results from phase 2 and 3 clinical trials using cetuximab or panitumumab alone or combined with chemotherapy in first-, second- or beyond-second-line treatment. The first meta-analysis reported that anti-EGFR agents did not significantly improve survival for *BRAF*-mt mCRC patients [nor PFS (HR, 0.88; *P* = 0.33) or OS (HR, 0.91; *P* = 0.63)] compared with standard chemotherapy or best supportive care.^42^ The second meta-analysis showed no significant interaction between anti-EGFR treatment and *BRAF* status for PFS and OS; the authors concluded that the *BRAF* mutation could not actually be considered as a negative predictive biomarker for anti-EGFR monoclonal antibodies in mCRC—that is, the presence of mutated BRAF should not preclude patients from receiving anti-EGFR therapy—and that further data are required to clarify this observation.^43^ Both these meta-analyses are subject to many limitations, and overall cannot guide our practice. First, not all available studies were included in these two meta-analyses; second, several lines of treatment with different populations and expected survival were mixed; third, negative trials for anti-EGFR agents with irrelevant backbone chemotherapeutic regimens (such as capecitabine plus oxaliplatin) were included; fourth, control arms mixed various chemotherapy regimens or even best supportive care; and fifth, both panitumumab and cetuximab trials were mixed although they might give different results in *BRAF*-mt patients. All these points are likely to present significant confounding factors when evaluating *BRAF*-mt mCRC patients.

Recently, a randomised phase 2 trial has evaluated the effect of adding panitumumab to triplet chemotherapy in first-line RAS wild-type mCRC patients. The addition of anti-EGFR agents to FOLFOXIRI improved the response rate in the whole study population of 96 patients (ORR, 85.7% versus 60.6%, *P* = 0.0096), without improving PFS (OS data not available). In a subgroup of *BRAF*-mt patients, the ORR also improved impressively (71% versus 22%), even though statistical significance was not reached, probably due to the limited number of *BRAF-*mt patients (*n* = 16).^44^

Although anti-EGFR agents do not confer any benefit to pre-treated *BRAF*-mt mCRC patients, these results suggest that they might be of value in the first-line treatment of such patients, especially if the goal of the treatment is tumour shrinkage. However, as stated above for anti-angiogenic therapies, trials comparing an aggressive chemotherapy ± an anti-EGFR therapy dedicated to *BRAF*-mt mCRC patients are still awaited. Finally, the FIRE-3 trial has compared FOLFIRI plus bevacizumab with FOLFIRI plus cetuximab in the first-line treatment of *RAS* wt mCRC patients. For the 48 (*n* = 14%) *BRAF*-mt patients identified in this trial, the ORR was higher in the cetuximab arm than in the bevacizumab arm (52% versus 40%), while no statistical differences were observed for PFS (HR, 0.84, *P* = 0.56) and OS (HR, 0.79, *P* = 0.45),^45^ suggesting that EGFR and VEGF inhibitors have equivalent therapeutic efficacy in BRAF-mt mCRC patients, except for response rate that favours anti-EGFRs.

### Targeting BRAF

*BRAF* mutations are found in many cancers and are particularly common in melanoma. In patients with V600E *BRAF*-mt metastatic melanoma, vemurafenib, a tyrosine kinase inhibitor specific to the ATP-binding domain of BRAF V600E, significantly improves both OS and PFS compared with dacarbazine, and facilitates response rates of 48% (versus only 5% with dacarbazine).^46^ However, the beneficial effect of BRAF-inhibitor monotherapy, using either vemurafenib or encorafenib, another ATP-competitive kinase inhibitor, seems much more limited in patients with *BRAF*-mt mCRC, with fewer than 10% of responders and PFS of 2.1–4.3 months.^47–50^ Based on these data, BRAF inhibitors alone seem to have insufficient clinical activity in patients with *BRAF* -mt CRC.

### Combining BRAF inhibitors and anti-EGFR agents

Preclinical studies conducted on *BRAF*-mt mCRC cell lines have shown that BRAF inhibition leads to the rapid feedback activation of EGFR, which could explain the persistence of tumour proliferation despite BRAF inhibition, as shown on Fig. 1b.^51^ Lower levels of EGFR expression by cancerous melanoma cells compared with CRC cells might explain the observed differences between melanoma and CRC in terms of response rates to BRAF-inhibitor monotherapy. Accordingly, the addition of cetuximab to encorafenib had a synergistic anti-proliferative effect in a human xenograft model of *BRAF*-mt CRC.^27^ In a pilot trial of 15 patients with *BRAF*-mt mCRC, the combination of vemurafenib and panitumumab induced modest anti-tumour activity. Tumour regression was seen in 10 of 12 patients, with partial responses in two patients (100 and 64% regression lasting 40 and 24 weeks, respectively) and stable disease lasting over 6 months in two others.^52^ In a basket trial, only one response was observed in the group of patients with mCRC who received vemurafenib combined with cetuximab, although tumour regression was observed in several other patients, albeit without fulfilling the RECIST 1.1 partial response criteria. Median PFS and OS values for these patients were 3.7 (95% CI, 1.8– 5.1) and 7.1 months (95% CI, 4.4 to not reached), respectively.^50^

More interestingly, when vemurafenib at different doses was combined with cetuximab and irinotecan in 17 *BRAF*-mt CRC patients in a phase 1b study, partial responses were observed in 35% of patients, with a median PFS of 7.7 months.^53^ The SWOG S1406 study then randomised 99 patients with *BRAF*-mt mCRC pre-treated with one or two lines of systemic chemotherapy to two arms of irinotecan plus cetuximab plus vemurafenib, with PFS as the primary objective.^54^ Median PFS was 4.4 months with the triplet therapy versus 2.0 months in patients treated with the doublet cetuximab plus irinotecan (HR, 0.42; *P* = 0.0002). Response rate and disease control rate (DCR) were also significantly higher for patients receiving the triplet drug combination (ORR, 16% versus 4%, *P* = 0.09; and DCR, 67% versus 22%, *P* < 0.001, respectively). Side effects were more common in the triplet arm, comprising mainly neutropenia, anaemia, nausea and arthralgia, and led to treatment discontinuation in 18% of cases. The subgroup analyses of this study should also provide more data about the efficacy of this triplet approach, especially in BRAF-mt MSI patients. Despite the limited number of patients included in the above-mentioned studies, this new strategy of double EGFR–BRAF inhibition shows undeniable signs of activity, and could represent a promising therapeutic option for *BRAF*-mt mCRC patients in the future.

### Combining BRAF inhibitors, anti-EGFRs and PI3K/AKT or MEK inhibitors

In preclinical studies, CRC cell lines also show high levels of PI3K/AKT pathway activation, which might contribute to resistance to BRAF-targeted monotherapy, as shown in Fig. 1d.^55^ In fact, the activation of this alternative pathway has already been described as a classical resistance mechanism to BRAF/RAS/MAPK pathway blockade. In *BRAF*-mt mCRC patients, a phase 1b trial has evaluated the therapeutic effect of encorafenib with cetuximab (doublet) ± alpelisib (an α-specific PI3K inhibitor) (triplet) in 28 patients.^56^ Best ORR and PFS were, respectively, 23.1% and 3.7 months (95% CI, 2.8–10.6) in the dual arm versus 32.1% and 4.3 months (95% CI, 4.1–5.4) in patients treated with the triplet, which seemed relatively well tolerated. The most common treatment-related grade 3/4 effects were fatigue and hypophosphataemia (8% each) in patients treated with the doublet, and hyperglycaemia (11%) and increased lipase (7%) in the triplet arm.

Combination strategies involving both MEK and BRAF inhibitors together with anti-EGFRs also significantly improved PFS in previously untreated melanoma patients.^57^ The combination of the BRAF inhibitor, dabrafenib, with panitumumab and the MEK inhibitor trametinib has also been tested with interesting results (ORR 26%, median PFS 4.1 months), with the limitation of significant skin toxicities.^57^ The combination of the BRAF inhibitor, dabrafenib, with panitumumab and the MEK inhibitor trametinib (ORR, 26%; median PFS, 4.1 months), with the limitation of significant skin toxicities.^58^

Thus, combining inhibition of EGFR and MAPK pathways with BRAF-targeted therapies together with a MEK inhibitor or with an action on the PI3K/mTOR alternative pathway using a PI3K inhibitor might be promising options to improve outcomes of *BRAF*-mt mCRC patients, and several trials are currently underway (Table 2). An open-label large phase 1 study has recently evaluated the triple combination of BRAF/MEK/EGFR inhibitors (as shown in Fig. 1c) in 142 patients with *BRAF-mt* CRC, and shows promising results (confirmed response rates of 21%) with an acceptable safety profile, with mostly dermatological toxicity.^51^ However, further randomised studies are required for a number of reasons: first, to find the most effective combination; second, to improve the tolerability of these combination therapies; and third, to compare them with standard chemotherapeutic regimens.

**Table 2:** Clinical trials involving *BRAF*-targeted therapies in BRAF-mt colorectal cancer patients

Therapeutic strategy	Regimen	*n*	ORR (%)	PFS (months)	Reference
BRAF inhibitor	Vemurafenib	21	5	2.1	48
	Vemurafenib	10	0	4.5	50
	Dabrafenib	9	11	NR	79
	Encorafenib	18	0	4	47
BRAF inhibitor + MEK inhibitor	Dabrafenib + trametinib	43	12	3.5	51
BRAF inhibitor + anti-EGFR mAb	Vemurafenib + cetuximab	27	4	3.7	50
	Encorafenib + cetuximab	26	19.2	3.7	80
	Encorafenib + cetuximab	50	22	4.2	81
	Vemurafenib + panitumumab	15	13	3.2	52
	Dabrafenib + panitumumab	20	0	3.5	51
BRAF inhibitor + anti-EGFR mAb + MEK inhibitor	Dabrafenib + panitumumab + trametinib	91	21	4.2	51
	Encorafenib + cetuximab + bimetinib	29	41	8	71
BRAF inhibitor + anti-EGFR mAb + PI3K inhibitor	Encorafenib + cetuximab + alpelisib	28	17.9	4.3	80
	Encorafenib + cetuximab + alpelisib	52	27	5.4	81
BRAF inhibitor + anti-EGFR mAb + CT	Vemurafenib + cetuximab + irinotecan	19	35	7.7	53
	Vemurafenib + cetuximab + irinotecan	54	16	4.4	54
	cetuximab + irinotecan	52	4	2	54

*ORR* objective response rate, *PFS* progression-free survival, *anti-EGFR mAb* anti-EGFR monoclonal antibody, *CT* chemotherapy

Further biomarker analyses will also be required to clarify the link between the genetic characteristics of the tumour and the response to treatment. Notably, combination strategies involving WNT pathway inhibitors in patients with *BRAF*-mt mCRC may be justified in the future by the observation of the association between *WNT5A* promoter methylation and *BRAF*^V600E^ mutation in CRC patients.^59^

## Immunotherapies

Targeting the immune system is a promising therapeutic option to improve the survival of some cancer patients, as shown in recent clinical trials involving immune checkpoint inhibitors in several tumour locations.^60,61^ However, studies evaluating immunotherapy in CRC patients, especially those using antibodies against programmed cell death protein 1 (PD1), have yielded disappointing results, with the exception of the subgroup of MSI patients, which is characterised by a strong immune infiltrate.^62^ Several studies have highlighted the overlap between the presence of *BRAF*^V600E^ mutations and MSI in CRC tumours.^7,15,63,64^ Indeed, *BRAF*-mt tumours are associated with the CpG island methylator phenotype (CIMP), which can lead to the inactivation of the MLH1 promoter, resulting in an MMR deficiency.^63^ In a pooled analysis of the CAIRO, CAIRO2, COIN and FOCUS studies involving primary tumours from 3063 patients, *BRAF* mutations were observed in 34.6% of patients with MSI tumours, whereas among *BRAF*-mt tumours 21.2% showed MSI.^65^ Higher correlation levels were found in a cohort study of 1253 patients, in which 52% of MSI tumours also had *BRAF* mutations, while 55% of the *BRAF*-mt tumours showed MSI.^66^

Given the encouraging results obtained in the MSI subgroup of CRC patients treated with PD1 inhibitors, it seems that there is an undeniable value in evaluating checkpoint inhibitors in the specific subgroup of MSI *BRAF*-mt patients. In addition, a positive correlation between the expression of programmed death ligand-1 (PD-L1) and the presence of mutated *BRAF*^V600E^ has been shown in *BRAF-mt* tumours, with higher levels of CD8^+^ tumour-infiltrating lymphocytes observed in *BRAF*-mt colorectal tumours,^67^ suggesting that *BRAF*-mt mCRC patients might benefit from immunotherapy.

In the CheckMate 142 trial, nivolumab, a checkpoint inhibitor targeting PD1, was tested in 74 pre-treated MSI mCRC patients, 12 (16%) of whom had *BRAF*-mt tumours. ORR and DCR for 12 weeks and more were, respectively, 31 and 69% versus 25 and 75% in *BRAF*-mt patients.^68^ Higher response rates were observed in the cohort of patients treated with nivolumab plus ipilimumab (a CTLA-4 inhibitor) (*n* = 119) in the same study, with an ORR of 55% and a DCR of 80% (median follow-up of 13.4 months). Interestingly, in *BRAF*-mt patients (*n* = 29), the ORR was not lower (55%) and the DCR > 12 weeks was 79%.^69^

Considering these results, it seems that immune checkpoint blockade may be more effective than BRAF-targeted therapies for *BRAF*-mt MSI mCRC patients. However, based on preclinical data that have shown an increase in the levels of both tumour antigens and the expression of major histocompatibility complex (MHC) molecules in patients treated with vemurafenib, combinations of immune checkpoint blockers and *BRAF*-targeted therapies are currently being tested in melanoma patients.^70^ This approach will need to be tested in the future for MSI*–BRAF*-mt mCRC patients.

## Ongoing studies

The BEACON study is the first multicentre, randomised, open-label, phase 3 three-arm study dedicated to *BRAF*-mt mCRC. The study compares, in mCRC patients pre-treated by one or two lines of treatment, the triplet encorafenib plus binimetinib (MEK inhibitor) plus cetuximab versus the doublet encorafenib plus cetuximab versus irinotecan plus cetuximab or FOLFIRI plus cetuximab (control arm) with OS as the primary objective in patients with *BRAF*-mt mCRC. After a median duration of follow-up of 18.2 months, results based on 29 patients with a *BRAF*^V600E^ mutation treated for a median duration of 5.6 months were promising, with an ORR of 48% (three complete and 11 partial responses), a median PFS of 8.0 months and a median OS of 15.3 months. Analysis of the safety lead-in cohort of the BEACON trial suggests an acceptable and manageable safety profile for patients receiving the encorafenib, binmetinib, and cetuximab combination. Dose-limiting toxicities occurred in five patients (including serous retinopathy and reversible decreased left ventricular ejection fraction) and were related to cetuximab-related infusion reactions for two of them.^71^ A very recent press release mentioned that the interim analysis of this study showed that the doublet (cetuximab + encorafenib) and the triplet (cetuximab + encorafenib + binimetinib) increased ORRs from 1.9% in the control arm to 20.4 and 26.1% in the experimental arms, respectively. OS was also improved in the two experimental arms with HR of 0.52 (95% CI, 0.39–0.70; *P* < 0.0001) and 0.60 (95% CI, 0.45–0.79; *P* *=* 0.0003). Full results of this interim analysis will be communicated in the forthcoming ESMO meetings.

Another phase 3 randomised trial designed to investigate FOLFOXIRI plus cetuximab or FOLFOXIRI plus bevacizumab as first-line treatment in *BRAF*-mt mCRC patients is currently underway, with a main objective of ORR (FIRE-4.5/AIO KRK-0116). Further phase 1/2 studies testing the efficacy and safety of combination therapies involving other BRAF inhibitors, PI3K, WNT and MEK inhibitors are currently ongoing and are summarised in Table 3.

**Table 3:** Ongoing studies in *BRAF*-mt patients according to National Cancer Institute registration

Therapy	Phase	Pts	Primary endpoint	Registration number
LGX818^a^ + cetuximab or LGX818 + BYL719^b^ + cetuximab	1/2	≥ L1	DLT PFS	NCT01719380
WNT974^c ^+ LGX818^a^ and cetuximab	1b/2	KRAS-wt ≥ L1	DLT ORR	NCT02278133
Encorafenib^a^, binimetinib^d^ and cetuximab	2	L1	Confirmed ORR	NCT03693170
Irinotecan + cetuximab + /− vemurafenib^a^	2	≥ L1	PFS	NCT02164916
FOLFOXIRI plus cetuximab or FOLFOXIRI plus bevacizumab	3	L1	ORR	FIRE 4.5 AIO KRK-0116
Encorafenib^a^ + cetuximab + /− binimetinib^d^ versus irinotecan/cetuximab or FOLFIRI/cetuximab	3	L2 L3	OS	BEACON NCT02928224

*OS* overall survival, *DLT* dose-limiting toxicities

^a^BRAF inhibitor; ^b^PI3K inhibitor; ^c^WNT inhibitor; ^d^MEK inhibitor

## Conclusion

The *BRAF*^V600E^ mutation is a major negative prognostic marker and is associated with resistance to standard chemotherapeutic regimens in mCRC patients, which justifies a personalised therapeutic approach in *BRAF*-mt mCRC patients. Although the best treatment has not yet been identified, an aggressive strategy involving triplet chemotherapy and a targeted therapy is currently the standard of care for fit patients. *BRAF*-targeted therapies have shown insufficient efficacy when used alone, but their combination with other targeted therapies such as anti-EGFRs, MEK inhibitors or PI3K inhibitors seems promising. Checkpoint inhibitors might also find their place in *BRAF*-mt mCRC patients with MSI, given the overlap between the *BRAF* mutation and the MSI phenotype. Finally, the place of each of the therapeutic combinations described and the way to sequence these new options remains an open question today. Further investigations are therefore justified, hence the need to promote the enrolment of *BRAF*-mt mCRC patients in clinical trials.

## Data Availability

Not applicable
